# Women Who Sell Sex in Eastern and Southern Africa: A Scoping Review of Non-Barrier Contraception, Pregnancy and Abortion

**DOI:** 10.3389/phrs.2022.1604376

**Published:** 2022-05-11

**Authors:** Catriona Ida Macleod, John Hunter Reynolds, Richard Delate

**Affiliations:** ^1^ Critical Studies in Sexualities and Reproduction, Rhodes University, Makhanda, South Africa; ^2^ Department of Sociology, Rhodes University, Makhanda, South Africa; ^3^ 2gether 4 SRHR, United Nations Population Fund, Johannesburg, South Africa

**Keywords:** pregnancy, sex workers, contraception, abortion, Eastern Africa, Southern Africa

## Abstract

**Objectives:** There is a need to hone reproductive health (RH) services for women who sell sex (WSS). The aim of this review was to collate findings on non-barrier contraception, pregnancies, and abortion amongst WSS in Eastern and Southern African (ESA).

**Methods:** A scoping review methodology was employed. Inclusion criteria were: 1) empirical papers from 2) ESA, 3) published since 2010, and 4) addressing WSS in relation to 5) the identified RH issues.

**Results:** Reports of rates of non-barrier contraceptive usage varied from 15% to 76%, of unintended pregnancy from 24% to 91%, and of abortion from 11% to 48%. Cross-cutting factors were alcohol use, violence, health systems problems, and socio-economic issues. Pregnancy desire was associated with having a non-paying partner. Barriers to accessing, and delaying, antenatal care were reported as common. Targeted programmes were reported as promoting RH amongst WSS.

**Conclusion:** Programmes should be contextually relevant, based on local patterns, individual, interpersonal and systemic barriers. Targeted approaches should be implemented in conjunction with improvement of public health services. Linked HIV and RH services, and community empowerment approaches are recommended.

## Introduction

Women who sell sex (WSS) receive money or goods in exchange for sexual services, either regularly or occasionally. Sex work varies in nature from formal and organised to informal. It constitutes consensual transactional sex between adults [1]. Given the nature of the work, it is unsurprising that WSS have special reproductive health (RH) needs [2]. For example, women who sell sex in sub-Saharan Africa are at higher risk of maternal morbidity and mortality than the general population because of their high rates of HIV, unintended pregnancies, and abortions [3].

The importance, thus, of honing sexual and reproductive health services to meet the needs of women who sell sex (WSS) is being increasingly recognised [4]. In 2014, Dhana and colleagues [5] published a review describing clinical and non-clinical facility-based sexual and reproductive health (SRH) services for WSS in Africa. The review revealed a narrow focus on HIV prevention, counselling and testing, and STIs; in addition, most interventions were localised and small-scale, operated with little coordination nationally or regionally, and had scanty government support. Broader SRH needs such as contraception services, antenatal care and abortion were generally ignored.

Women who sell sex in Africa generally experience limited economic options, many dependents, marital disruption, and low levels of education. Their work may involve violence, criminalisation, high mobility and hazardous substance use [6]. These factors, together with the occupational contexts of their work, have highlighted their vulnerability to HIV, about which a reasonable amount of research has been conducted [7]. Less, however, is known about WSS in relation to their reproductive health needs and desires. The aims of the review are to identify the following issues in relation to reproductive health amongst WSS in Eastern and Southern Africa: non-barrier contraceptive usage prevalence, and associated factors; unintended pregnancy prevalence and associated factors, pregnancy desires, antenatal care; abortion prevalence and associated factors; access and barriers to services; and positive service delivery programmes.

We take a reproductive health rights approach in this paper. The World Health Organization’s constitution envisages “the highest attainable standard of health as a fundamental right of *every* human being” [8] (emphasis added). A reproductive health rights approach means that states should ensure access to timely, acceptable, and affordable health care of appropriate quality. Such an approach is essential in designing broad-based programmes to address the reproductive health needs of marginalised communities, such as WSS.

## Methods

### Scope

The scoping review methodology employed by Arksey and O’Malley [9] was used in this project. This consists of the following stages: 1) identifying the research question; 2) identifying relevant studies; 3) study selection; 4) charting the data; and 5) collating, summarizing and reporting the results. This method is designed to systematically map the subject field.

### Publication Selection and Data Extraction

The following electronic databases were searched in April 2021: Academic Search Premier; Health Source: Nursing/Academic Edition; Medline; PsyArticles; PsyINFO; SocIndex; Sabinet; Web of Science; PubMed; and Google scholar. The keyword search for studies was: Female sex workers^1^ OR sex workers AND contraception OR family planning OR reproduct* OR pregnan* OR antenatal care OR abortion AND [list of countries] OR Eastern Africa OR Southern Africa. The search was restricted to the last 10 years to ensure that the information is current. No language restriction was placed on the search, in case there were relevant papers in another language (most likely French or Portuguese). The search, however, only surfaced papers written in English.

**FIGURE 1:**
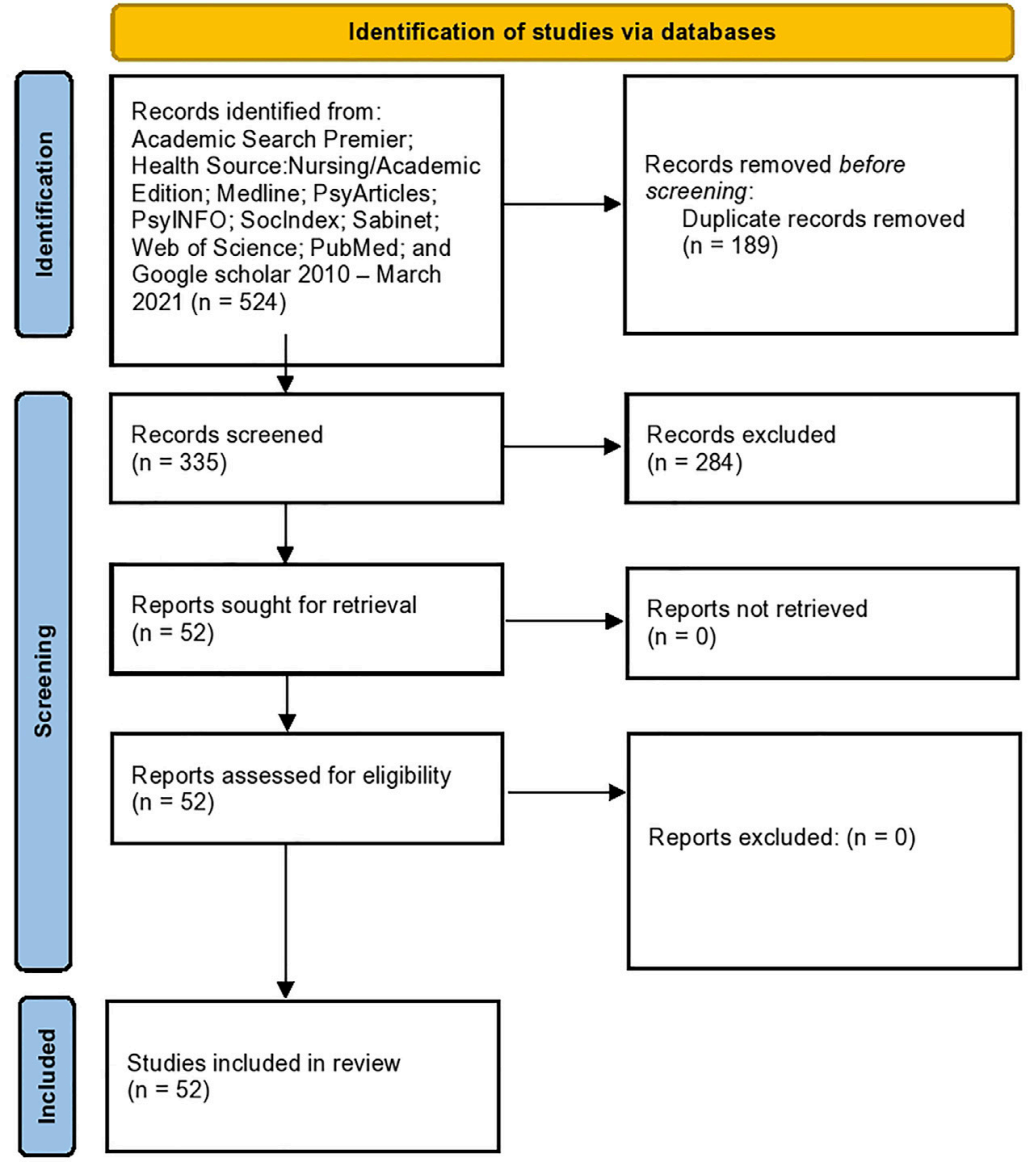
PRISMA diagram, Women Who Sell Sex in Eastern and Southern Africa: A Scoping Review of Non-Barrier Contraception, Pregnancy and Abortion (scoping review, Eastern and Southern Africa, 2010-2020).

The initial search produced 524 papers. After duplicates were removed, the two authors went through the papers independently, determining whether the identified studies were relevant to the research aims. Inclusion criteria were that the papers should: 1) be empirical papers; 2) specifically address WSS in relation to 3) the identified RH issues and 4) be conducted in ESA countries. Each author’s assessments were compared. Differences were resolved through discussion. The papers were quality checked through use of the Mixed methods appraisal tool (MMAT) [5]. No studies were discarded following this assessment. No further papers were found on checking the reference list of downloaded papers. The result was 53 papers. The process is displayed in an adapted PRISMA flow diagram in Figure 1.

The studies were charted as follows. First, eligible studies were summarized, including the following information for each publication: author(s); year of publication; study location; programme researched (where relevant); study populations; aims of the study; data collection method; important results; study recommendations. Second, attention was paid to the distribution of the studies in relation to country and SRH issue (non-barrier contraception, pregnancy, antenatal care, abortion). Third, the literature and information on programmes was organized thematically using the research aims provided above as a template for analysis.

## Results

Fourteen of the studies were conducted in Kenya [10–23], eight in South Africa [13, 14, 24–29], eight in Uganda [30–37], six in Mozambique [13, 14, 38–40], five in Tanzania [41–45], three in Eswatini [46–48] and in Malawi [49–51], two in Ethiopia [52, 53], in Zambia [54, 55], and in Zimbabwe [56, 57] and one each in Democratic Republic of Congo [58], Lesotho [59], Rwanda [60], and online (Australia, Brazil, El Salvador, France, Kenya, Malawi, Russia, South Korea, Spain, Tanzania, the United States of America, and Zimbabwe) [56]. Thus, knowledge production concerning WSS in relation to non-barrier contraception, pregnancy and abortion is dominated by studies conducted in Kenya. There are many countries in which no published studies have been conducted. Non-barrier contraception usage was the most researched topic. In contrast, there were few studies concentrating specifically on abortion.

In the following, we outline findings in relation to non-barrier contraception, pregnancies, abortion, needs and barriers in relation to health services, and promising programme developments.

### Non-Barrier Contraception

Table 1 provides an overview of the major findings and recommendations of studies that concentrated specifically on non-barrier contraceptive usage.

**TABLE 1:** ^a^Studies’ findings and recommendations for women who sell sex and non-barrier contraception (Women Who Sell Sex Project, Eastern and Southern Africa, 2010–2021).

CitationLocationProgramme	Study aims	Major relevant findings	Study recommendations
Study population	Data collection		
Abaasa et al. (2019)	To investigate reliable contraceptive use at baseline and 6 months in key-populations	Reliable methods of contraception were used by FSW = 179 (62%) which included 67% of women using injectable contraception, 14.8% using an implant, 14% using oral pills, 3% using an intra- uterine devices and 1% women sterilized. Women aged 18–34 years were twice as likely to use a reliable method compared to those aged 35 years or more	Promotion and provision of reliable contraceptives is needed
Uganda			
N/A	Survey		
FSWs and fisherfolk			
Ampt et al. (2018)	To assess correlates of long- acting reversible contraceptive (LARC) use, and explore patterns of LARC use among female sex workers (FSWs) in Kenya	The prevalence of contraceptive use was 22.6% for implants and 1.6% for intra-uterine devices (IUDs). LARC use was independently associated with previous pregnancy (adjusted odds ratio for one pregnancy), positive attitude to and better knowledge of family planning, younger age, and lower education. High rates of adverse effects were reported for all methods	Further intervention is required to improve both uptake (particularly of IUDs) and greater access to family planning services
Kenya			
N/A	Baseline survey questionnaire		
FSWs			
Bukenya et al. (2019)	Determine contraceptive use, the prevalence, and predictors of pregnancy planning among FSWs in Uganda	Of the 819 study participants, only 90 (11.0%) had planned pregnancies. Dual contraception use (condom and other modern method) was 58.0%. Having a non-emotional partner as a man who impregnated the FSW compared to emotional partner was significantly associated with less planned relative to unplanned pregnancy, so was lack of reported social support compared to support from friends	There is an urgent need to promote dual contraception among FSWs to prevent unplanned pregnancies especially with non-emotional partners, drug users, and post- rape
Uganda			
MARPI clinics	London Measure of Unplanned Pregnancy (LMUP) questionnaire		
FSWs from clinics			
Chanda et al. (2017)	Evaluate the prevalence of and factors associated with contraceptive use, unplanned pregnancy, and pregnancy termination among FSW in three transit towns	Of 946 women eligible for this analysis, 84.1% had been pregnant at least once, and among those 61.6% had an unplanned pregnancy, and 47.7% had a terminated pregnancy. Incarceration was associated with decreased odds of dual contraception use and increased odds of unplanned pregnancy. Condom availability at work was associated with increased odds of using condoms only for contraception and decreased odds of unplanned pregnancy	Increasing availability of condoms in the work place may be a low-cost intervention to improve condom use and improve reproductive health outcomes for FSW.
Zambia			
N/A	Survey questionnaire		
FSWs in transit towns			
Dulli et al. (2021)	Test an intervention to increase non-condom, modern method and dual method use among FSWs attending health services at drop-in centres (DIC) in two Kenyan cities	The intervention had a significant positive effect on non-condom, family planning method use, but no effect on dual method use. FSW reported both paying and non-paying partners also influence non- condom contraceptive use	Integrated services providing convenient access to family planning, HIV counselling and testing, and screening, diagnosis and treatment of other STIs may better address the sexual and reproductive health needs of FSW.
Kenya			
Experimental intervention	Two-group pre- and post-test quasi-experimental study: questionnaires		
FSWs			
Erickson et al. (2015)	To describe the characteristics of female sex workers (FSWs) who do and do not use dual contraceptives (i.e. male condoms plus a non- barrier method) in Gulu, northern Uganda	Among the 400 FSWs who participated, 180 (45.0%) had ever used dual contraceptives. In the multivariate model, dual contraceptive use was positively associated with older age, prior unintended pregnancy, and HIV testing. Having to rush sexual negotiations owing to police presence was negatively associated with dual contraceptive use	Integrated links between HIV and sexual health programs could support contraceptive uptake among FSWs
Uganda			
N/A	Survey questionnaire		
FSWs			
Faini et al. (2020)	Explore FSWs’ pregnancy perceptions and experiences of unintended pregnancy	FSWs reported that sex work impedes good contraceptive behaviour because sex workers felt unable to negotiate consistent condom use, avoided health services due to stigma, missed monthly contraceptive supplies because of inconvenient clinic operating hours or skipped contraceptive pills when intoxicated after taking alcohol. FSWs who perceived pregnancy to be a burden terminated the pregnancy because of fear of loss of income during pregnancy or child rearing expenses in case child support was not assured by their partners. FSWs who perceived pregnancy to be a blessing decided to keep the pregnancy because they desired motherhood and hoped that children would bring prosperity	Findings underscore the need to integrate contraceptive services with HIV programs serving FSWs in their areas of work
Tanzania			
N/A	In-depth individual interviews		
FSWs			
Ingabire et al. (2019)	Assess impact of anonymous HIV counselling and testing, diagnosis and treatment of STIs and long-acting reversible contraception (LARC) to FSWs in Kigali, Rwanda	From September 2012 to March 2015, 1168 FSWs sought services, including 587 (50%) who were HIV- positive. Modern contraceptive use was reported by 43% of HIV-positive and 56% of HIV-negative FSWs (*p* < 0.0001). Current pregnancy was reported by 4% of HIV-positive and 6% of HIV-negative FSWs (*p* = 0.0409)	Tailored and integrated HIV/STIs and family planning programs are urgently needed for FSWs
Rwanda			
HIV counselling and testing, diagnosis and treatment of STI and long-acting reversible contraception (LARC)	Clinical data		
FSWs in hotspots			
Kilembe et al. (2019)	The aim of the study was to compare reproductive health and high-risk behaviours in female sex workers (FSWs) and single mothers (SMs) in Zambia’s two largest cities, Lusaka and Ndola	From 2012 to 2016, 1,893 women (1,377 FSWs and 516 HIV- SMs) responded to referrals. In all groups, consistent condom use (8%–11%) and modern contraceptive use (35%–65%) were low	Tailored and targeted reproductive health services are needed to reduce HIV, STI, and unplanned pregnancy in these vulnerable women
Zambia			
N/A	Tests results of HIV/STIs		
FSWs and single mothers			
Lafort et al. (2016)	Use of, and barriers to, HIV and sexual and	The cross-sectional survey showed that 71% of FSWs used non-barrier contraception, 55% sought	Access to, and use of, HIV and SRH services should be
Mozambique, Zimbabwe	reproductive health (HIV/SRH) commodities and services for female sex workers (FSWs) were assessed as part of a baseline situational analysis	help at a health facility for their last unwanted pregnancy. Local public health facilities were by far the most common place where care was sought, followed by an NGO-operated clinic targeting FSWs, and places outside the Tete area. In the focus group discussions, FSWs expressed dissatisfaction with the public health services, as a result of being asked for bribes, being badly attended by some care providers, stigmatisation and breaches of confidentiality. The service most lacking was said to be termination of unwanted pregnancies	improved by reducing barriers at public health facilities, broadening the range of services and expanding the reach of the targeted non- governmental (NGO) clinic
DIFFER	Survey questionnaire		
FSWs			
Long et al. (2019)	Evaluate the prevalence and predictors of unmet contraceptive need in HIV- positive FSWs	Among 346 HIV-positive FSWs, 125 (36.1%) reported modern non-barrier contraceptive use, leaving 221 (63.9%) with unmet contraceptive need. Condom use was the only form of contraception for 129 (37.3%) participants. In unadjusted analyses, unmet contraceptive need was associated with physical abuse in the past year by someone other than a regular partner, desire for (more) children, and having 2–3 previous pregnancies compared to 0–1 prior pregnancies. In adjusted analyses, lower number of previous pregnancies and having desire for future children remained significantly associated with a higher prevalence of unmet contraceptive need	These findings highlight the need for concerted efforts to identify and eliminate barriers to contraceptive use in FSWs living with HIV.
Kenya			
N/A	Survey questionnaires		
HIV-negative FSWs			
Mbita et al. (2020)	To examine protection against STIs/HIV and unintended pregnancy (dual method use) among FSWs in an outreach-based HIV prevention, care, and treatment program in Tanzania	119,728 FSWs made a first visit to services served by the Sauti Project from January 2016 to September 2017. Of these 79,774 were current contraceptive users—of those, 4548 (5.7%) took a contraceptive as well as condoms, the study measure of dual family planning (FP) method use. Ninety-one percent (n = 4139) of FSWs taking dual FP methods were provided with an injectable in addition to condoms. Dual method use was lower in this study than in research studies in the region, highlighting potential differences between findings from research studies and evidence from a routine service provision setting	The findings call for further research and programs to address FSW agency to increase dual protection against STIs/HIV and unintended pregnancy
Tanzania			
Sauti	Programme surveillance data		
FSWs			
Ochako et al. (2018)	To explore the experiences of female sex workers with using existing contraceptive methods, assess individual and health facility-level barriers and document inter- partner relationship in the use of contraceptives	Findings reveal that while some FSWs know about modern contraceptives, others have limited knowledge or out rightly refuse to use contraceptives for fear of losing clients. The interaction with different client types act as a barrier but also provide an opportunity for contraceptive use among FSWs. Most FSWs recognize the importance of dual protection for HIV/STI and pregnancy prevention. However, myths and misconceptions, fear of being tested for HIV at the family planning clinic, wait time, and long queues at the clinics all act in combination to hinder uptake of contraceptives	A targeted approach to address the contraceptive needs of FSWs to help remove barriers to contraceptive uptake. The introduction of counselling services to provide information on the benefits of non-barrier contraceptive methods and thereby enhance dual use for both pregnancy and STI/HIV prevention
Kenya			
N/A	Focus group discussions		
FSWs			
Sibanda et al. (2021)	To explore contraceptive values and preferences among sex workers	Survey participants reported an awareness of modern contraceptive methods. FGDs found that younger women had lower awareness. Reports of condomless sex were common and modern contraceptive use was inconsistent. Determinants of contraceptive choices included ease of use, ease of access to a contraceptive method, and fewer side	Although in the study sex workers have good awareness of contraceptives, this does not translate into good access, choice, and use. health coverage which leaves no one behind
Zimbabwe and online			
N/A			
FSW	Online survey questionnaire; interviews; focus group discussions	effects. Healthcare provider attitudes, availability of methods, and clinic schedules were important considerations. Most sex workers are aware of contraceptives, but barriers include male partners/clients, side effects, and health system factors such as access and clinic attitudes towards sex workers	
Schwartz et al. (2017)	Consider comprehensive family planning needs among FSW, including the demand for preconception services, across three sub- Saharan African countries	Overall 1666 FSW were enrolled, 1372 (82.4%) of whom had ever been pregnant. Twenty-five per cent of FSW had an unmet need for contraception; 9% of FSW employed dual contraception, including highly effective non-barrier methods and consistent condom use. Nineteen per cent (*n* = 313/1666) of FSW were trying to conceive. HIV-positive, undiagnosed FSW were more likely to be trying to conceive as compared to HIV-negative FSW; among 98 HIV-positive women trying to conceive, 25.5% were on antiretroviral therapy	FSW have varying reproductive goals and contraceptive usage. Efforts to improve coverage of comprehensive family planning – including efforts to increase HIV testing and engagement in treatment among FSW trying to conceive – are necessary for the prevention of mother to child transmission
Eswatini (Swaziland), Burkina Faso and Togo			
N/A	Questionnaire; HIV testing		
FSWs			
Slabbert et al (2017)	Understand how the sexual and reproductive health (SRH) status of female sex workers is influenced by a wide range of demographic, behavioural and structural factors	Only about 15% of women in both sites were using modern contraceptives. Johannesburg women were also more likely to access health services at a hotel (85.0% vs. 80.6%) or clinic (5.7% vs. 0.5%), to have completed secondary education (57.1% vs. 36.0%), and moved house more than twice during the past year (19.6 vs. 2.0%)	Segmenting sex worker populations according to age, country of origin and place of service delivery, and training healthcare providers accordingly, could increase uptake of SRH services
South Africa			
N/A	Records of FSWs		
FSWs			
Srivatsan et al. (2019)	To study contraceptive usage and ARV treatment by FSWs in Lesotho	56% of HIV + participants were not using non-barrier contraception..	Tailored HIV information delivery efforts for FSW
Lesotho			
N/A	Survey questionnaire		
FSWs			
Sutherland et al. (2011)	Document patterns of contraceptive use and unmet need for contraception	The reported level of modern contraceptives in the setting was very high. However, like in other studies, there was a great reliance on male condoms, coupled with inconsistent use at last sex, which resulted in a higher potential for unmet need for contraception than the elevated levels of modern contraceptives might suggest. Dual method use was also frequently encountered in this population and the benefits of this practice were clearly outlined by focus group participants	These findings suggest that the promotion of dual methods among this population could help meet the broader reproductive health needs of FSWs
Kenya			
N/A	Survey; focus group discussions		
FSWs			
Twizelimana and Muula (2021)	Estimate the prevalence of unmet contraceptive needs and examined associated factors among FSWs	Out of the 290 study participants 102 (35.2%) reported unmet contraceptive needs. The following factors were significantly associated with unmet contraceptive needs in multivariate analysis: female sex workers’ history of physical and sexual violence by clients, participants with a steady partner, and participants who feared side effects of contraceptives	Reproductive Health services should address barriers to contraceptives use. There is need to improve awareness of contraceptives. Specific health promotion interventions on female sex workers engaged in a steady partnership are recommended
Malawi			
N/A	Survey questionnaires		
FSWs			
Twizelimana and Muula (2020)	Investigate the actions taken by FSWs after condom failure among	Out of 18 FSWs who experienced condom failure, 10 reported to have stopped sex immediately and changed the condom and then resumed afterwards	Health programs should develop interventions and support the performance of
Malawi	FSWs in semi-urban, Blantyre in Malawi	They reported to have douched, urinated, and/or squatted to prevent pregnancy, sexually transmitted infections (STIs) and HIV acquisition. 10 FSWs didn’t seek medical care. They thought the actions taken were enough for HIV and pregnancy prevention. Out of the 18 FSWs, only 3 stopped sexual intercourse completely and sought medical care which included post-exposure prophylaxis for HIV, STI treatment, and emergency contraceptives	safer sex and actions after condom failure among FSWs to prevent STIs including HIV, and unplanned pregnancies
N/A	Focus group discussions and in-depth individual interviews		
FSWs			
Yam et al. (2014)	Examine emergency contraceptive pills (ECP) use among FSW in Swaziland	In weighted analyses, 27.5% of FSW had ever used ECP. Most (77.8%) had ever been pregnant, among whom 48.7% had had an unwanted pregnancy and 11.7% had had an abortion. Significant independent correlates of ECP use were younger age, higher education, higher income, having two or more children, and never having been married	Older and poorer FSWs may not have adequate access to ECP.
Eswatini			
N/A	Survey questionnaire		
FSWs			
Yam et al. (2013)	Understand sex workers’ use of condoms and non- barrier methods	After adjustments were made for background and behavioural factors, 16% of female sex workers were found to be consistent users of condoms alone; 39% used non-barrier modern methods (without consistent condom use); 8% were dual method users; and 38% were inconsistent condom users or used other methods or none. Respondents who had children were more likely than their nulliparous counterparts to report use of non-barrier methods alone (65% vs. 14%	Inconsistent or no condom use among non-barrier contraceptive users underscores the need to incorporate HIV prevention into family planning interventions, particularly among female sex workers who have children and non-commercial partners
Eswatini			
N/A	Survey questionnaire		
FSWs			

a In the tables we use the terms used by the authors. Thus, mostly, women who sell sex are referred to as female sex workers.

Reports of the use of non-barrier modern contraception varied considerably across studies. The lowest was 15% in a South African study [24]. The highest was 76.3% in one site in a Kenyan study [10]. The former study accessed records of WSS attending regular services across two cities, while the latter reported on baseline data of a targeted intervention in towns where tourists, migrant workers and military personnel have attracted a high number of WSS. This may account for the differences noted. One study was conducted in the context of a targeted intervention (in Rwanda), and reported usage of 43% by HIV-positive participants and 56% by HIV-negative participants [19]. The rest of studies, using respondent-driven survey data or longitudinal data, reported usage somewhere between 30% and 71%: 36.1% and 30.5% in two Kenyan studies [11, 16]; 39% in a study conducted in Eswatini [13]; between 35% and 41% in a Zambian study [54]; 47.5% in a different site in the above-mentioned Kenyan study [14]; 56% amongst HIV-positive WSS in Lesotho [59];; 66.6% in Zambian study [55]; 71% in a study conducted in Mozambique [38].

A study focussing on long-acting reversible contraception in Kenya found that 22.6% of participants used implants and 1.6% IUDs. Dual contraception usage was reported as low in some studies—5.7% in the context of a targeted programme in Tanzania [41]; 8% in Eswatini (respondent-driven survey) [46]; 9% in Eswatini (combined study with Togo and Burkina Faso—respondent driven survey) [48]. However, others reported higher rates—43.4% in a Malawian study (systematic sampling survey) [49], 58% in a Ugandan study (survey in context of targeted services) [30], 30.7% and 50.5% in two sites in Kenya (baseline for targeted service) [10]; 38% in another Kenyan study (respondent-driven survey) [17]. Only one Eswatini study (respondent-driven survey) focused on emergency contraception: 27.5% of study participants had ever used emergency contraception [47].

Various studies addressed variables associated with non-use of non-barrier contraceptive. Studies did not necessarily use the same variables. We therefore list all found (noting that some may not apply in certain areas, while others may apply but were not included in the study). Variables include: personal factors – fear of side effects [10, 51]. desire for (more) children [11], being nulliparous [46], history of incarceration or arrest [55], intoxication [42], and being older than 35 [31]; interpersonal factors—male partners’ or clients’ disapproval [10], physical or sexual abuse [9, 16], having a steady partner [51]; and systemic issues—poor clinic access [10, 42], negative healthcare provider attitudes [10], and condom availability at work [55]. Use of non-barrier contraception was found in to be associated with ease of access, positive healthcare provider attitudes, conducive clinic schedules, fewer side effects [56], previous pregnancy, positive attitude to and knowledge of family planning, younger age, and lower education [18]. Independent correlates of emergency contraception use were younger age, higher education, higher income, having two or more children, and never having been married [47]. Dual contraception was positively associated in a Ugandan study with older age, prior unintended pregnancy and HIV testing. Rushing sexual negotiations owing to police presence was negatively associated with dual contraception usage [32]. In a qualitative study conducted in Kenya, Ochako and colleagues [19] found that most participants recognised the importance of dual contraception but that there were various barriers to use, including misconceptions (e.g. IUDs falling out), fear of being tested for HIV at family planning clinics, wait times and long queues.

### Pregnancy and Antenatal Care

Table 2 outlines the studies’ findings and recommendations in relation to unintended pregnancies, pregnancy desire, and pregnancy care.

**TABLE 2:** Studies’ findings and recommendations for women who sell sex and pregnancy (Women Who Sell Sex Project, Eastern and Southern Africa, 2010–2021).

CitationLocationProgramme	Study aims	Major findings	Study recommendations
Study population	Data collection		
Beckham et al. (2015)	Explore FSWs’ experiences with intended pregnancy and access to antenatal care and HIV testing in two regions of Tanzania	FSWs sought to become pregnant to gain respect as mothers, to avoid stigma, and/or to solidify relationships, sometimes posing risks to their own and their partners’ health. Pregnant FSWs generally sought antenatal care (ANC) services but rarely disclosed their occupation, complicating provision of appropriate care. Accessing ANC services presented particular challenges, with health care workers sometimes denying all clinic services to women who were not accompanied by husbands. Several participants reported being denied care until delivery. The difficulties participants reported in accessing health care services as both sex workers and unmarried women have potential social and health consequences in light of the high levels of HIV and STIs among FSWs in sub-Saharan Africa,	Reproductive health services, including but not limited to ANC and PMTCT, must be tailored to fit FSWs’ unique contexts. The health system could benefit from sensitization training for health care workers and national guidelines for health care services for FSWs. Community mobilization interventions can reduce stigma and increase women’s willingness to disclose their occupation to health care workers and to demand their rights to health care and other services
Tanzania			
N/A	In-depth individual interviews		
FSWs			
Bukenya et al. (2019)	Evaluate the psychometric properties of the London Measure of Unplanned Pregnancy (LMUP) among female sex workers (FSWs)	Concluded that the Luganda LMUP is a valid and reliable tool for assessing pregnancy planning among FSWs in Uganda and that the Acholi, Lugisu, and Runyankole versions of the LMUP also had good initial psychometric properties	Using the LMUP with FSWs can be an alternative method to the other ways of assessing unplanned pregnancies such as in the DHS. The LMUP can be used to evaluate and refocus interventions to reduce unplanned pregnancies among FSWs in Uganda
Uganda			
The Most at Risk Population Initiative (MARPI) clinics	LMUP questionnaire		
FSWs from clinics			
Duff et al. (2017)	Examine the correlates of unintended pregnancies among young women sex workers in conflict-affected northern Uganda	Among 400 sex workers (median age = 20 years), 175 (43.8%) reported at least one unintended pregnancy. In multivariable analysis, primarily servicing clients in lodges/brothels, hormonal contraceptive usage and drug/alcohol use while working were positively correlated with previous unintended pregnancy	These findings highlight a need for improved access to integrated reproductive health and HIV services, catered to sex workers’ needs. Sex work-led strategies (e.g., peer outreach) should be considered, alongside structural strategies and education targeting brothel/lodge owners and managers
Uganda			
N/A	Survey questionnaire		
Young FSWs			
Lokken et al. (2020)	To describe the incidence and correlates of pregnancy in HIV-positive Kenyan sex workers	Two hundred seventy-nine FSWs were eligible (October 2012-April 2017). Most women had a non- paying, regular partner (83.2%, 232/279), were not using modern non-barrier contraception (69.5%, 194/279), and did not desire additional children (70.6%, 197/279). Of 34 first incident pregnancies, 91.2% (*n* = 31) were unintended. The incidences of planned were similar. In univariable analysis, oral contraceptive pill use (versus no contraception), having a non-paying, regular partner, transactional sex, vaginal washing, condomless sex, and higher sex frequency were associated with an increased pregnancy risk. Older age was associated with a lower pregnancy risk. In multivariable analysis, having a non-paying, regular partner and age ≥40 years remained	In the context of comprehensive care for HIV-positive FSWs, regular ascertainment of fertility desires and pregnancy intentions could increase effective contraceptive use in women not trying to conceive and facilitate uptake of safer conception strategies for pregnancy planners
Kenya			
N/A	Monthly questions to ascertain sexual behaviour and quarterly pregnancy testing		
Current and former FSWs living with HIV			
		significantly associated with a higher and lower pregnancy risk, respectively	
Luchters et al. (2016)	Determine the rate, predictors and consequences of unintended pregnancy among FSWs	Four hundred women were enrolled, with 92% remaining in the cohort after 1 year. Fifty-seven percent reported using a modern contraceptive method (including condoms when used consistently). Over one-third (36%) of women were using condoms inconsistently without another method. Twenty-four percent had an unintended pregnancy during the study. Younger age, having an emotional partner and using traditional or no contraception, or condoms only, were independent predictors of unintended pregnancy. Women attributed pregnancy to forgetting to use contraception and being pressured not to by clients and emotional partners, as well as "bad luck". They described numerous negative consequences of unintended pregnancy	Reproductive health services need to be incorporated into programs for sexually transmitted infections and HIV, which address the socially- determined barriers to contraceptive use. Providing contraception information and addressing barriers to contraception uptake through mobile phones could offer a new way to reach and engage FSWs
Kenya			
N/A	Quarterly quantitative data collection via structured questionnaire and testing for pregnancy and HIV; focus group discussions and in- depth interviews with FSWs who became pregnant during the study, and interviews with five key informants		
FSWs			
Parmley et al. (2019)	Explore pregnancy and post- delivery experiences of mothers who practice sex work	FSWs experienced and feared violence by clients during pregnancy, highlighting the need for safe work environments. Further, FSWs expressed concerns about HIV acquisition and vertical transmission during the perinatal period. Physical challenges related to pregnancy affected women’s ability to work. Returning to work post-delivery presented barriers to initiating and practicing exclusive breastfeeding. As a result, many FSWs practiced mixed feeding	These data highlight the need for integrated SRHR services for FSWs, including PMTCT services. Mentor mother programs, tailored for FSWs, may also provide an opportunity for improved infant health outcomes in this context
South Africa			
	In-depth individual interviews		
FSWs			
Parmley et al. (2019)	Characterize factors influencing ANC seeking behaviors in a high HIV prevalence context	In the quantitative survey, 77% of FSW were mothers (313/410); of these, two-thirds were living with HIV (212/313) and 40% reported being on antiretroviral therapy (ART) (84/212). FSW in the qualitative sub- sample reported unintended pregnancies with clients due to inconsistent contraceptive use; many reported discovering their unintended pregnancies between 4 and 7 months of gestation. FSW attributed delayed ANC seeking and ART initiation in the second or third trimesters to late pregnancy detection. Other factors limiting engagement in ANC included substance and alcohol use and discontent with previous healthcare- related experiences	Integrating comprehensive family planning services into FSW programming, as well as providing active linkage to ANC services may reduce barriers to accessing timely ANC, decreasing risks of vertical transmission
South Africa			
N/A	Pregnancy and HIV testing; in-depth individual interviews		
FSWs			
Rao et al. (2016)	Assess the association between human immunodeficiency virus (HIV) and pregnancy intentions and safer conception knowledge among female sex workers	Overall 391 women were represented in the analyses. More than 50% had a prior HIV diagnosis, and an additional 12% were diagnosed with HIV during the study. Approximately half (n5185) of the women reported future pregnancy intentions. In univariate analysis, a prior HIV diagnosis was negatively associated with pregnancy intentions as compared with HIV-negative women. Only parity remained independently associated with future pregnancy intentions in multivariate regression after controlling for HIV status, age, race, relationship status, and years selling sex. Knowledge of safer conception methods such as timed sex without a condom, preexposure prophylaxis, or self-insemination was low and similar between those with and without future pregnancy plans	Findings suggest a need to provide female sex workers with advice around options to conceive safely in the context of high HIV prevalence
South Africa			
N/A	Questionnaire; HIV testing		
FSWs			
Twizelimana and Muula (2020)	Investigate the correlates of pregnancy among FSWs	The prevalence of pregnancy was 61% for FSWs born in rural place as compared to 37% for those who were born in town. In multivariate analysis FSWs who reported to value being respected as mothers had 12 times the risk of pregnancy comparing to the ones who did not. FSWs who reported using condoms inconsistently had five times the risk of pregnancy compared to the ones who did not. FSWs who had a request to bear children from steady partners had 5 times the risk of pregnancy comparing to the ones who did not. FSWs who reported forgetfulness of contraceptives’ use had 3 times more risk of pregnancy comparing to the ones who did not	There is a need for access to reproductive health services integrated in antiretroviral therapy (ART) programs. It is important to recognize the child bearing desires and circumstances of FSWs in order to inform health programs responsive to their needs
Malawi			
FSWs	Questionnaire; interviews		
Weldegebreal et al. (2015)	Assess unintended pregnancy and associated factors among female sex workers	The magnitude of unintended pregnancy among female sex workers in the past 2 years was 28.6%. During this period, 59 women had abortion which represents three-fifths, (59.6%), of those who had unintended pregnancies, and 17.1% of all female sex workers. Female sex workers who gave birth and had history of abortion formerly had 3.1 and 15.6 times higher odds of unintended pregnancy compared to their counterparts, respectively. Sex workers who had steady partners had 2.9 times higher odds of have unintended pregnancy than those who hadn’t. Drug users had 2.7 times higher odds of unintended pregnancy than those who hadn’t use. Sex workers who had 60–96 months of duration in sex work were 67% less likely to have unintended pregnancy than those with <12 months)	Ongoing and continuous counseling on safe sex, including correct and consistent use of condom and, for particular clients, enhancing use of emergency contraceptive methods will benefit to reduce unintended pregnancy among FSWs. Tailored strategies and mechanisms should be developed to address unintended pregnancy and its consequences
Ethiopia			
FSWs	Survey questionnaire		
Wilson et al. (2018)	Investigate fertility desire in HIV-positive female sex workers	The effect of fertility desire on PSA detection varied significantly by non-barrier contraception use. At visits when women reported not using non-barrier contraception, fertility desire was associated with higher risk of semen detection. However, when women used non-barrier contraception, fertility desire was associated with lower risk of PSA detection. Fertility desire was not associated with detectable VL or higher absolute risk of transmission potential	Low HIV transmission potential regardless of fertility desire suggests that the combination of condoms and antiretroviral therapy adherence was effective
Kenya			
N/A	Standardized face-to-face interviews; clinical data: prostate specific antigen (PSA); detection of semen and STIs; detectable plasma viral load (VL)		
FSWs			
Yam et al. (2020)	Describe fertility intentions, need for contraception, and awareness of, or interest in safer conception services; and examine the characteristics associated with desire to have a child imminently	Nearly one-third wished to have a child within 2 years. Seventy-two percent had heard of having the HIV-positive partner taking ART to reduce sexual transmission during pregnancy attempts. Thirty-one percent felt the amount of FP content covered in the consultation was “too little.” Factors significantly associated with desire for children were having a non- paying partner and having fewer children. Viral suppression was not associated with fertility desire	Sex workers living with HIV attending integrated HIV/FP services have need for both contraception as well as safer conception counselling. FP counselling for HIV-positive women should be broadened to broach the topic of safer pregnancy, as well as explicit counselling on strategies to minimize risk of sexual transmission to partners
Tanzania			
Sauti	Exit interviews		
FSWs			
Yam et al. (2017)	Examine the circumstances surrounding pregnancy and childbirth among women selling sex	The women reported on pregnancies experienced both before and after they had begun selling sex. They identified some of the fathers as clients, former partners, and current partners, but they did not know the identities of the other fathers. Missed injections,	Though they represent a small proportion of the population, the holistic sexual and reproductive health needs of FSWs should be met in a coordinated, integrated
Ethiopia			
Link up project			
FSWs	In-depth individual interviews	skipped pills, and inconsistent condom use were causes of unintended pregnancy. Abortion was common, typically with a medication regimen at a facility. Comprehensive sexual and reproductive health services should be provided to women who sell sex, in recognition and support of their need for family planning and their desire to plan whether and when to have children	fashion, with an emphasis on upholding their fundamental right to plan whether and when to have children

Yam et al. [53] outline the challenges in meeting the reproductive health needs of pregnant sex workers, these being: “an entrenched societal aversion regarding FSWs [female sex workers] as pregnant women or mothers, the “siloed” nature of HIV and reproductive health programming and financing, and the challenges of balancing FSWs’ disease prevention needs with the childbearing desires” (p. 117).

The question of unintended pregnancies was addressed in a number of studies, with reports of rates varying. For example, the following rates were reported in Kenyan studies:

• Of those with first pregnancies in [16] study, 91.2% were reported as unintended.
• In a Sutherland’s study [17], unintended pregnancies were reported by 52% of participants.
• Luchters et al. [20] found that 24% of their Kenyan participants had an unintended pregnancy during the study conducted over 12 months

In a Zambian survey, Chanda et al. [55] report that of the respondents who had been pregnant, 61.6% had had an unplanned pregnancy. In Northern Uganda, Duff et al. [33] found an unintended pregnancy rate of 43.8%. None of these rates were collected in the context of a study about targeted services.

Factors associated with unintended pregnancies were: primarily servicing clients in lodges or brothels [33]; hormonal contraceptive (injections) usage [33]; drug or alcohol use during work [33, 52]; having four or more living children [34]; non-emotional partner as a man who fathered last pregnancy [34]; having had an abortion [34]; being unmarried [34]; having a steady non-paying partner [52]; and longer duration of sex work [52].

Twizelimana and Muula [50] emphasise the importance of considering the child bearing desires and circumstances of women who sell sex so that health programmes can respond to their needs. Non- paying partner request and being born in a rural area contributed to pregnancy desire for their Malawian participants. This is confirmed in a Tanzanian study [43]. in which just under one-third of participants desired having a child in the next 2 years. Having non-paying partners and fewer children were associated with this desire.

In South African study [25], about half the participants reported future pregnancy intentions. In univariate analysis, HIV diagnosis was negatively associated with pregnancy intentions as compared with HIV-negative women. But in multivariate analysis, only parity remained independently associated with future pregnancy intentions. In a three country study (Eswatini, Burkina Faso and Togo), Schwartz et al. [48] found that HIV-positive, undiagnosed sex workers were more likely to be trying to conceive than HIV-negative sex workers. Wilson et al. [21] noted that fertility desire could increase HIV transmission in HIV-positive sex workers. However, in their Kenyan study, they found that the combination of condoms and antiretroviral therapy adherence was effective in preventing this.

Knowledge of safer conception methods was investigated in two studies. In a South African study [25], 59.3% of women knew of ARV-based methods for safer conception, and 14.3% of non-ARV methods. In Tanzania [43], 90% of participants knew of one safer conception method, with 72% having heard of having the seropositive partner taking ART.

Delayed seeking of antenatal care was found in a South African study [27]. This was attributed to late pregnancy detection, alcohol and substance use, and discontent with previous healthcare-related experiences. Challenges accessing antenatal care were likewise revealed in a Tanzanian study [44]. Healthcare workers reportedly would sometimes deny clinic services to women not accompanied by their husbands. In addition, participants indicated that they rarely disclosed their occupation to healthcare workers, thereby jeopardising receiving appropriate care.

In a qualitative study conducted in Gqerbherha (formerly Port Elizabeth) [26], South Africa, sex workers indicated that they had experienced violence by clients during pregnancy. They expressed concerns about HIV acquisition and vertical transmission of HIV during the perinatal period. Physical challenges during pregnancy affected their ability to work, and work post-partum interfered with exclusive breast- feeding.

### Abortion

Only three studies in the dataset concentrated specifically on abortion. This is regrettable, given the fact that, as pointed out by Marlow and colleagues [35], sex workers’ need for safe abortion services is greater than that of other women of reproductive age because of their number of sexual contacts, and their increased risk of sexual violence. The major findings and recommendations from these studies are contained in Table 3.

**TABLE 3:** Studies’ findings and recommendations for women who sell sex and abortion (Women Who Sell Sex Project, Eastern and Southern Africa, 2010–2021).

CitationLocationProgramme	Study aims	Important results	Study recommendations
Study population	Data collection		
Chareka, Crankshaw and Zambezi (2021)	Explore the range of SRHR needs and challenges amongst YWSS (16–24 years)	Our findings indicate that abortions occur amongst YWSS in Zimbabwe but there remain questions over the extent of safety of abortions. The restrictive legal context around abortion and illegality of sex work in the country are key determinants underlying the clandestine nature of abortions. Socioeconomic concerns are key in decision-making around abortions. Youth, cost and lack of referral networks contribute towards unsafe abortions, even when safe abortion services are available. Many YWSS are not aware of the availability of post abortion care (PAC) services and resort to self-administered PAC. Being young and selling sex combine and interact on the economic and social levels to produce vulnerabilities greater than their sum to experiencing unsafe abortion	Greater efforts need to be made at the national level to offer services that are not only safe in terms of quality of care but also that are viewed as safe to access for young women already experiencing high levels of stigma and discrimination and who are disproportionately burdened by poor SRH outcomes
Zimbabwe			
N/A	Focus group discussions; in-depth individual interviews		
Young women who sell sex (YWSS), peer educators, health care providers and key informants			
Erickson et al. (2017)	Explore factors associated with lifetime abortions among FSWs; model the independent effect of lifetime exposures to incarceration and living in internally displaced persons (IDP) camps on coerced and unsafe abortions	Of 400 FSWs, 62 had ever accessed an abortion. In a multivariable model, gendered violence, both childhood mistreatment/or abuse at home and workplace violence by clients were linked to increased experiences of abortion. Lifetime exposure to incarceration retained an independent effect on increased odds of coerced abortion, and living in IDP camps was positively associated with unsafe abortion	These results suggest a critical need for removal of legal and social barriers to realising the SRH rights of all women, and ensuring safe, voluntary access to reproductive choice for marginalised and criminalised populations of FSWs
Uganda			
N/A	Data collection		
FSWs			
Marlow et al. (2014)	Understand sex workers’ experiences with induced abortion services or post- abortion care (PAC) at an urban clinic in Uganda	Five women came to the clinic for post-abortion care (PAC) and four women came for an induced abortion. All but one of the women had children, with an average of two children each (range: 1–4). Four of the nine women dropped out of school when they were in primary school or the first year of secondary school. The other women did not mention their level of educational attainment. All of the women seeking PAC services at the clinic took a local herb to induce abortion at home before arriving at the clinic. Four women took the herb ennanda and one of the women who took ennanda also took the herb oluwoko. The fifth woman who took local herbs said that the woman supplying the local herb would not tell her the name of the herb. Two women who came to the clinic for an induced abortion were advised by friends to take local herbs, but the women instead decided to come to the clinic for induced abortion and had not taken any herbs	Findings point to creating community-level interventions in which women can speak openly about abortion, creating a support network among sex workers, training peer educators, and making available a community outreach educator and community outreach workshops on abortion. At the health facility, it is important for service providers to treat sex workers with care and respect, allow sex workers to be accompanied to the health facility and guarantee confidentiality
Uganda			
N/A	In-depth individual interviews		
FSWs seeking induced abortion or post-abortion care services			

A number of studies do, however, refer to abortion in passing, confirming Marlow et al.’s assertion. A survey of WSS in Eswatini found that 48.7% had had an unwanted pregnancy, and 11.7% had undergone an abortion [47], while in an Ethiopian study [52], 59.6% of participants with an unintended pregnancy had an abortion, or 17.1% of all participants. Chanda et al. [55] report that of the sex workers who had been pregnant at least once in their Zambian survey, 47.7% had terminated an unplanned pregnancy. In a Kenyan study [10], 17.5% and 12.8% of respondents in two sites indicated that they had had an abortion. Participants in Lafort et al.’s [38] study in Mozambique reported that the most lacking service was for the termination of unwanted pregnancies. Sex workers in Tanzania reported terminating their pregnancies because of fear of loss of income during pregnancy or because of child rearing expenses [42].

Reasons for seeking abortions included not knowing the man responsible for the pregnancy, inability to raise an additional child, incest, wanting to continue with education [35], and socioeconomic concerns [57]. Gendered violence, including childhood maltreatment at home and workplace violence by clients were associated in a Ugandan study with abortions.

In a Zimbabwean study [57], youth, cost and lack of referral networks were reported as contributing to unsafe abortions, even when safe abortion services were available. Awareness of post abortion care (PAC) services was low, resulting in women having self-administered PAC. Marlow et al. [35] found in their Ugandan study that women took a local herb to induce abortion. The lower cost of taking herbs often swayed women in their decision. Erickson et al. [36] found that incarceration and living in internally displaced persons camps were associated with coerced and unsafe abortions respectively. 37.

### Health Services Needs and Barriers

Table 4 outlines major findings and study recommendations for studies focussing specifically on health services needs and barriers for women who sell sex.

**TABLE 4:** Studies’ findings and recommendations for women who sell sex and health services (Women Who Sell Sex Project, Eastern and Southern Africa, 2010–2021).

CitationLocationProgramme	Study aims	Important results	Study recommendations
Study population	Data collection		
Afzal, Lieber and Beddoe (2020)	Understand regional barriers and attitudes regarding reproductive health care needs	Community discussion groups revealed a desire for easier and more accessible healthcare, showing the biggest barriers to care are lack of money and transportation, and safety concerns related to profession, including fear of violence from partner and/or client	Fostering community ownership sets the stage for future implementation of sustainable and cooperative health programming
South Africa			
N/A	Focus group discussions; survey questionnaire		
FSWs			
Corneli et al. (2016)	Identify barriers to accessing contraceptive services among FSWs and preferences for contraceptive service delivery options among FSWs and health care providers (HCPs)	Three barriers were identified that limited the ability of FSWs to access contraceptive services: (1) an unsupportive clinic infrastructure, which consisted of obstructive factors such as long wait times, fees, inconvenient operating hours and perceived compulsory HIV testing; (2) discriminatory provider–client interactions, where participants believed negative and differential treatment from female and male staff members impacted FSWs’ willingness to seek medical services; and (3) negative partner influences, including both non- paying and paying partners. Drop-in centers followed by peer educators and health care facilities were identified as preferred service delivery options	Alternative delivery options, such as drop-in centers coupled with peer educators, may be an approach worth evaluating
Kenya			
N/A	Focus group discussions		
FSWs and healthcare providers			
Gichuna et al. (2020)	Highlight specific effects of COVID-19 and related restrictions on healthcare access for the sex workers	Existing gender and health inequalities have been reinforced by the initial outbreak of the COVID-19 pandemic. The various restrictions imposed made it difficult for the sex workers to access their healthcare needs. There was a shortage of family planning options and even where available, some of the sex workers could not access them	Sex worker organisations could be involved in providing COVID- 19 testing and contact tracing among sex workers. One of the positive elements of the Covid crisis is that NGOs have had to respond flexibly to the needs of their service users
Kenya			
N/A	In-depth individual interviews		
FSWs in informal settlements; healthcare providers			
Kiernan et al. (2016)	Explore the experience of urban sex workers	Analysis identified several themes: (1) economic hardship as a catalyst for joining the sex trade, (2) significant work- related violence and (3) a paucity of available resources or assistance. Responses to specific prompts indicated that sex workers do not trust law enforcement and there are significant barriers to both medical care and local resources	Further studies of this vulnerable population and its needs are encouraged in order to develop programmes that provide the means to manage the hazards of their work and obtain an alternative source of income
Democratic Republic of Congo			
N/A	In-depth individual interviews		
FSWs			
Lafort et al. (2016)	Use of, and barriers to, HIV and sexual and reproductive health (HIV/SRH) commodities and services for female sex workers (FSWs) were assessed as part of a baseline situational analysis	The cross-sectional survey showed that 55% sought help at a health facility for their last unwanted pregnancy. Local public health facilities were by far the most common place where care was sought, followed by an NGO-operated clinic targeting FSWs, and places outside the Tete area. In the focus group discussions, FSWs expressed dissatisfaction with the public health services, as a result of being asked for bribes, being badly attended by some care providers, stigmatisation and breaches of confidentiality. The service most lacking was said to be termination of unwanted pregnancies	Access to, and use of, HIV and SRH services should be improved by reducing barriers at public health facilities, broadening the range of services and expanding the reach of the targeted NGO clinic
Mozambique, Zimbabwe			
DIFFER	Survey questionnaire		
FSWs			
Lafort et al. (2016)	A baseline cross-sectional survey to measure where	Across cities, FSWs most commonly sought care for the majority of HIV/SRH services at public health	The best model to improve access, linking targeted
Kenya, Mozambique, South Africa, India	FSWs seek HIV/SRH care and what motivates their choice	facilities, most especially in Durban. Services specifically targeting FSWs only had a high coverage in Mysore for STI care (89%) and HIV testing (79%). Private-for-profit clinics were important providers in Mombasa, but not in the other cities. The most important reason for the choice of care provider in Durban and Mombasa was proximity, in Tete ‘where they always go’, and in Mysore cost of care. Where available, clinics specifically targeting FSWs were more often chosen because of shorter waiting times, perceived higher quality of care, more privacy and friendlier personnel	interventions with general health services, will need to be tailored to the specific context of each city
DIFFER	Survey questionnaire		
FSWs			
Makhakhe et al. (2019)	The aim of this study was to understand the functioning of non- governmental health care services as well as to document the experiences of FSWs utilising these services	The FSWs expressed challenges related to SRH care access at public health facilities. The majority felt that they could not consult for SRH-related services because of stigma. The non-governmental health and advocacy organisations providing SRH services to FSWs through their mobile facilities utilising the peer approach, have done so in a way that promotes trust between FSWs and mobile health care providers. FSWs have access to tailored services, prevention materials as well as health information. This has resulted in the normalising of HIV testing as well as SRH seeking behaviours	In its quest for health care reform, the South African health sector should engage with these organisations and aim to design government-led parallel services that have a wider reach, and with sensitised health care staff so as to gradually cater for key populations
South Africa			
NGO services	Focus group discussions and in-depth individual interviews		
FSWs			
Robert et al. (2020)	Identify enablers and barriers in access of HIV and sexual reproductive health (SRH) services among adolescent key populations (KP) in Kenya	Adolescent KPs preferred to access services in private health due to increased confidentiality, limited stigma and discrimination, access to adequate amount of condoms, friendly and fast- tracked services. Negative health provider attitudes made adolescent KPs dislike accessing health care in public health facilities. There was a lack of adolescent key population’s policies and guidelines on HIV and SRH.	Identify enablers and barriers in access of HIV and sexual reproductive health (SRH) services among adolescent key populations (KP) in Kenya
Kenya			
N/A	Focus group discussions; in-depth individual interviews		
Adolescent WSS and drug injectors			

A number of barriers to contraceptive services were identified in the studies. These include: long clinic wait times [22]; having to pay medical fees [22, 28]; being asked for bribes [38]; inconvenient clinic operating hours [22, 42]; perceived compulsory HIV testing at clinics [22]; discriminatory provider-client interactions [22]; inadequate care [38, 58]; paucity of available services [58]; stigmatisation [29, 38, 42]; breaches in confidentiality [38]; lack of transport [28]; negative partner influences [22, 28].

Gichuna and colleagues [23] conducted interviews with WSS and healthcare practitioners in Kenya regarding service delivery during the COVID-19 pandemic. Unsurprisingly, they found that the sex workers struggled to access services, and “to accept the [resultant] harsh reality of carrying unwanted pregnancies” (p. 1430).

Lafort et al. [38] found that 55% of women who sell sex in Tete, Mozambique sought help at a health facility for their last unwanted pregnancy. Public health facilities were the most frequently used, followed by an NGO-operated clinic targeting sex workers. Likewise, in a Kenyan study [12], it was found that young WSS prefer accessing services in private healthcare on the basis of better confidentiality, limited discrimination and stigma, adequate commodities, and fast and friendly services. Drop-in centres and peer educators were identified as preferred service delivery options. Healthcare resources and service coverage in general are key issues in SRH services for WSS. By way of example, Lafort etal [39]. note that in the area in which they conducted their research, Tete, Mozambique, basic services were available, but not certain contraception methods and termination of pregnancy. Public facilities face serious challenges in terms of space, staff, equipment, regular supplies and adequate provider practices. Private clinics offer some services, but at commercial prices.

In a study of healthcare preferences of sex workers in Kenya, South Africa and Mozambique, Lafort et al. [13] found that the most common factors in choice of care provider (most often public health facilities) was proximity and familiarity. Where targeted services were available, they were chosen because of the shorter waiting times, perceived quality of care, more privacy and friendlier personnel.

### Positive Service Delivery

In this section, we report on studies outlining the development and implementation on services that show promise. The Diagonal Interventions for Fast-Forward Health (DIFFER) is a programme developed and piloted in India, Kenya, South Africa and Mozambique. It was aimed at improving targeted services for WSS and public health services, as well as cooperation between the two. Findings from a qualitative evaluation of the DIFFER intervention in Mozambique showed a significant increase in non-barrier contraceptive usage, with this increase being attributed to the WSS-targeted outreach rather than utilization of public health clinics [61]. While some public health facilities were reported to be WSS- friendly, barriers such as stock-outs, bribery, and disrespectful treatment remained in many. Lafort et al. [14] report that in all cities in which DIFFER was implemented, the uptake of services increased – from 12.5% to 41.5% in Durban, 25%–40.1% in Tete, and 44.9%–69.1% in Mombasa. In Tete and Mombasa, the rise in SRH service use was almost entirely due to greater uptake of targeted services. It was only in Durban that there was an increase in public health facility use.

In a different paper, Lafort et al. [40] reflect on the feasibility of up-scaling the DIFFER programme. Interviews with key informants—policymakers, government employees, international development or NGO workers and community representatives – revealed that expansion of targeted services were hampered by financial constraints, institutional capacity and lack of buy-in. In addition, making existing public services friendlier to key populations was preferred to the targeted approach.

Makhakhe and colleagues [29] report a similar finding concerning targeted services in South Africa. Participants felt that they could not consult public SRH services because of stigma. Instead, non-governmental health and advocacy organisations providing SRH services through mobile facilities or through peer interactions were seen as promoting trust and providing tailored services. The authors caution, however, that these services are provided in urban areas, leaving those outside of these sites vulnerable to the health risks associated with a lack of access of tailored services.

Ampt et al. [15] report on a randomised control trial that tested the efficacy of a multifaceted short messaging service intervention concerning contraceptive knowledge and behaviours (WHISPER) in reducing unintended pregnancies. When compared to the control (SHOUT—nutrition focused messages), the intervention had no measurable effect on unintended pregnancies (15.5 per 100 person-years compared to 14.7 per 100 person-years). They argue that, when used in isolation, these kinds of interventions will not have a significant impact on unintended pregnancies amongst sex workers.

Service delivery for WSS has largely concentrated on the prevention of HIV. These services, however, can have an effect on non-barrier contraceptive usage as well. For example, in an investigation of the effect of the Shikamana HIV programme in Tanzania, Kerrigan et al. [45] found increases in the use of modern contraception in follow-up visits.

Rosenberg et al. [37] outline the findings from a pilot programme targeted at refugee women who sell sex in Uganda. They indicate that taking a community empowerment approach can facilitate access to a range of critical information, services and support options in these circumstances. This approach includes information on how to use contraceptives, referrals for friendly HIV testing and treatment, peer counselling and protective peer networks.

Gichuna and colleagues [23] emphasise the importance of innovative approaches to supporting the health of WSS. For instance, their partner NGO, Bar Hostess Empowerment and Support Programme (BHESP), uses online platforms and phone technology to deliver peer information, advice and advocacy for sex workers; this is being enhanced to reach women who are mobile and transient. The phone app is paired with a flexible outreach model, using a motorcycle to deliver essential medications to WSS.

## Discussion

Higher rates of contraceptive usage may be expected in studies reporting on targeted services. However, reportage of non-barrier contraceptive usage and unintended pregnancies were not associated with whether the study reported on a targeted service. Thus, thee variability of rates of contraception usage and unintended pregnancies points to the need for contextually relevant programmes based on knowledge of local usage patterns and needs, including rates amongst the general population, which also varies considerably: lifetime contraceptive usage in sub-Saharan Africa varies from 30% to 76% [62].

Dual contraception and emergency contraception can greatly reduce the incidence of unintended pregnancies. But use of dual contraception, for the most part, and emergency contraception, where reported, was shown to be low. Future pregnancy intentions were associated with having a non-paying partner, but not with HIV status. Knowledge of safer conception varied. Lack of referral networks and living in displaced persons camps were associated with unsafe abortion. Incarceration was associated with coerced abortion. Awareness of post-abortion care was reportedly low.

Reports of unintended pregnancies vary across the studies, but for the most part are higher than the average rate of reported unintended pregnancies across sub-Saharan Africa, which stands at 29% [63]. It is important, however, that country specific rates be considered in comparisons. For example, Luchters et al. [20] describe the rate of unintended pregnancies found in their Kenyan study (24%) as high. However, Mumah et al. [64] of the Kenyan Population Council indicate that “Levels of unintended pregnancy among Kenyan women have changed little over the last 5 years, declining from 45 percent in 2003 to just 43 percent in 2008/09” (non-paginated) [The latest Kenyan Demographic and Health Survey does not list the prevalence of unintended pregnancies]. In this case, thus, the participants in Luchters et al.’s study had lower rates of unintended pregnancies than did the general population of women of reproductive age.

In Table 5, we consolidate the findings regarding factors relating to non-use of non-barrier, dual and emergency contraception, unintended pregnancies, delayed antenatal care, and abortion. It should be noted that some of the factors featured may apply in other areas, but were not featured in the studies under review. Varying factors refer to where the direction of the factor (e.g., older or younger age) is not consistent across the RH areas. Consistent factors are where the direction of the factor is the same across the areas. Unique factors feature in only one area (as outlined in the studies reviewed). Alcohol and substance use and abuse featured across three areas, violence over two, poor health systems over three, and socio-economic issues over two. This suggests that addressing alcohol use, violence, health systems problems across programmes may bear fruit, along with tackling poverty.

**TABLE 5:** Varying, consistent and unique factors (Women Who Sell Sex Project, Eastern and Southern Africa, 2010–2021).

Varying, consistent and unique factors associated with					
Non-use of non-barrier contraception	Non-use of dual contraception	Non-use of emergency contraception	Unintended pregnancy	Delayed antenatal care	Abortion
**Varying: age-related**					
>35 year old	Younger age	Older age			Wanting to continue with education
**Varying: pregnancy related**					
Being nulliparous	No prior unintended pregnancy		Having had an abortion	Late detection of pregnancy	
**Varying: child related**					
Desire for more children		Having only one child	Having four or more living children		Inability to raise another child
**Varying: Partner related**					
Male partner or clients’ disapproval; Having a steady partner		Being married	Non-emotional partner as man who fathered last pregnancy; Having steady non- paying partner; Being unmarried		Not knowing man responsible for pregnancy
**Consistent: alcohol or substance use**					
Intoxication			Drug or alcohol use during work	Alcohol and substance abuse	
**Consistent: Violence**					
Physical or sexual abuse					History of violence; violence in the workplace; Incest
**Consistent: Health system issues**					
Poor clinic access; Poor healthcare provider attitudes	Wait time and long queues at clinics; fear of being tested for HIV at family planning clinics			Discontent with previous healthcare experiences	
**Varying: Workplace issues**					
Condom availability at work			Servicing clients in lodges or brothels; Longer duration of sex work		
**Consistent: socio-economic**					
		Lower income and lower education			Socio-economic concerns
**Unique factors**					
History of incarceration or arrest	No prior HIV testing; fear of HIV testing		Using hormonal injections		
Fear of side effects	Rushing sexual negotiations				
	Misconceptions				

Many barriers to services, probably exacerbated by COVID, were recorded. Some of these are systemic (e.g., long wait time, operating hours, paucity of available services), while other have to do with the service providers’ actions (e.g., WSS being asked for bribes, discrimination and stigmatisation against WSS). Some WSS reported experiencing violence by clients during pregnancy. Pregnancy affected their ability to work, while work negatively affected childcare.

Increase in non-barrier contraception usage was recorded in a diagonal intervention (targeted services together with improved public health services - DIFFER), but was attributed mainly to the targeted services. Focussed provision and promotion of non-barrier contraceptive methods in a simulated HIV vaccine efficacy trial led to significant increases in use.

### Public Health Implications of Findings

Recommendations emanating from this review are as follows:

• Context is important in terms of designing services; this includes site of sex work, available public services, geographical location, and demographics of sex workers (e.g. age, mobility).
• Targeted approaches, including peer educators, outreach services, mobile clinics, phone apps, seem to bear fruit; given financial constraints in implementing these to scale, however, concurrent improvement of public health facilities’ services and access is important.
• Training of healthcare and social service providers in a rights-based approach to RH amongst WSS is important. This includes the creation of safe WSS friendly care.
• Counselling for safe conception and early pregnancy detection for women who desire children should be provided.
• Integrated links between HIV and sexual and reproductive health programmes should be created to support contraceptive uptake.
• Mentor mother programmes tailored for WSS should be developed.
• Programmes should address misconceptions regarding services (e.g., automatically being tested for HIV) and commodities (e.g., fear of contraceptive side effects).
• Community empowerment approaches are encouraged, including use of peer educators and the creation of joint strategies to reduce violence.
• Awareness of legal abortion services (within parameters allowed within the country), and of post-abortion care should be raised.
• Content and activities that are accessible to mobile WSS should be developed.
• Guidelines for accessing and servicing adolescent WSS should be developed.
• Alcohol and substance abuse, health system problems, and violence featured across a number of RH areas explored in this paper. This suggests that programmes tackling these issues may bear fruit.

In addition, research in under-served areas (e.g., rural areas), in countries in which there has been little or no knowledge production, and in relation to abortion is needed to flesh out context specific, comprehensive and relevant services.

### Strengths and Limitations

This paper brings together the findings of many studies in relation to reproductive health amongst WSS. As such, it contributes to ongoing efforts to ensure that the reproductive rights of WSS are realised. It provides a road map for states as well as non-governmental organisations to ensure access to timely, acceptable, and affordable health care of appropriate quality, in line with a reproductive rights approach.

There are, however, some limitations to this paper. Women who sell sex are a hard to reach population. Many of the studies reported on in this review drew on samples presenting at clinics, respondent-driven sampling, or convenience sampling. Possibilities for generalisation are thus limited. Additionally, studies were conducted in diverse contexts; caution should be taken in applying the findings across the region.

### Conclusion

In line with a reproductive rights approach, women who sell sex are entitled to reproductive health services. This review has highlighted the multiple barriers that WSS experience in relation to these services, as well as the factors involved in non-use of contraception, unintended pregnancies, delayed antenatal care, and abortion. This information, along with studies showing what and how programmes targeted at WSS work, should be used to improve the reproductive health and rights of WSS in Eastern and Southern Africa.

## Footnotes

1 Note of terminology: most public health publications use the term “female sex workers”. In this paper we prefer the person-first approach, using, line with others [65].
